# On the move: understanding home care workers’ experiences of using various modes of transportation at work in an occupational health perspective

**DOI:** 10.1186/s12913-024-12071-z

**Published:** 2024-12-18

**Authors:** Ingeborg Frostad Liaset, Marius Steiro Fimland, Svend Erik Mathiassen, Skender Redzovic

**Affiliations:** 1 Department of Neuromedicine and Movement Science, Faculty of Medicine and Health Sciences, NTNU Norwegian University of Science and Technology, 7491 Trondheim, Norway; 2 Unicare Helsefort Rehabilitation Centre, 7112 Rissa, Norway; 3 Department of Occupational Health, Psychology and Sports Sciences, Centre for Musculoskeletal Research, University of Gävle, 801 76 Gävle, Sweden

**Keywords:** Home health worker, Health care worker, Bicycle, Electrical scooter, Transport

## Abstract

**Background:**

The demand for home care workers (HCWs) is increasing, but home care services face challenges in recruiting and retaining skilled workers, partly due to hazards in the work environment. Transportation to client visits is an important part of HCWs' working conditions, with various modes (e.g., walking, cycling, driving) being utilized. However, these modes are often implemented without considering HCWs' perceptions of their use. Therefore, our study aimed to understand HCWs’ perceptions and experiences of using different transportation modes at work, and how they may influence health.

**Methods:**

Fourteen HCWs from a home care unit in Trondheim (Norway) participated in focus group interviews. The interviews were analyzed using a reflexive thematic analysis approach including reflexive journaling. The analytical process was guided by a biopsychosocial understanding of health.

**Results:**

The analysis showed that when different transportation modes were assigned, predictability of the assignment was important for the HCWs. Both walking and driving were regarded to have both positive and negative health impacts. When walking, informants thought that getting fresh air outdoors and doing physical activity was health-promoting, while bad weather conditions and too much walking could be negative for their health. When driving a car, informants talked about privacy and getting physical rest as positive for their health, while traffic and parking conditions could be stressful. Individual factors such as age, physical health, and strong preferences were highlighted as important to consider when planning HCWs’ transportation modes in an occupational health perspective.

**Conclusions:**

Walking now and then between client visits was generally believed by the HCWs to lead to positive health effects compared to only driving a car. Introducing planning of various transportation modes in advance, so that they are predictable, seems important to reduce stress among HCWs. In addition, some individual factors should be considered in the planning, and it should be realized that the planning likely represents a trade-off between promoting the psychosocial work environment when driving a car and potentially enhancing long-term physical health when using active transportation. Thus, biopsychosocial aspects of health should be considered when planning the mode of transport between client visits for HCWs.

**Supplementary Information:**

The online version contains supplementary material available at 10.1186/s12913-024-12071-z.

## Background

The demographic composition of populations in the Western world, including Norway, is changing towards a larger proportion of elderly, while political decisions at the same time attempt to move health care from institutions to primary care [1, 2]. Home care services facilitating that elderly can live in their own home are essential in this context, in offering a cost-effective way to maintain citizens’ independence and quality of life [3, 4]. Thus, the need for home care workers (HCWs) is increasing. At the same time, Norwegian home care services are struggling to recruit and retain qualified HCWs [5, 6]. This may partly be explained by physical and psychosocial hazards in the work environment, which affect the health of HCWs negatively [7]. HCWs report to have high workloads, a high level of strain, and a low work autonomy, and they describe their work as stressful, physically demanding and exhausting [3, 8–10]. Thus, the prevalence of work-related musculoskeletal pain, headache and psychological exhaustion among HCWs in Norway is high compared to other occupational sectors [10, 11]. Increasing the knowledge of working conditions affecting the health of HCWs is imperative, both to ensure a sustainable, high-quality home care service [4, 12], and to increase the attractiveness of working in home care.

The workday of HCWs differ from that of health care workers in institutions since HCWs provide assistance to citizens living in their own home, and therefore spend a considerable amount of time on transport between locations [13, 14]. Thus, HCWs in Norway spend up to 30% of their total working time on transportation [1, 15], and the conditions during transportation may well influence HCWs’ attitudes to stay in the job [16, 17]. However, very few previous studies have been devoted to documenting the HCWs’ experiences of transportation during the workday [17].

In urban municipalities, HCWs may use different modes of transportation, i.e., cars, electrical or regular bicycles, electrical scooters, or walking [18]. Traditionally, research has focused on time-use when investigating transportation in home care services [1, 15, 19, 20], with an increasing number of studies modelling the use of different transportation modes in order to develop a more effective use of home care resources [21]. For instance, Szander et al. [19] found that a combination of transportation modes (i.e., bus, walking, electrical bicycle, car) showed a potential for decreasing total time used on transportation in urban districts, and that combining different transportation modes is therefore a more sustainable choice in home care work than using only cars. This may be one explanation that combined transportation has been implemented in urban Norwegian municipalities.

Few studies have investigated the use of different transportation modes in home care from an occupational health perspective [22]. These studies indicate that using active transportation in home care work increases HCWs’ time spent in physical activity, but the studies have not reported on HCWs’ experiences when using different transportation modes at work, and how they perceive that different transportation modes might influence their health. While some studies briefly touch upon HCWs’ perceptions of driving a car in relation to their occupational health [7, 23], more extensive studies of HCWs’ perspectives of using different transportation modes at work seem to be lacking. Studies are therefore needed to understand HCWs’ perceptions and experiences of using different transportation modes at work, and how they may influence health. In the present study, HCWs’ perceptions and experiences of using different transportation modes at work were investigated to provide a deeper understanding of how transportation modes may influence health. For this purpose, we used a reflexive thematic analysis of focus group discussions with HCWs.

## Methods

### Research context

The Norwegian home care service is publicly funded and provides nursing care and practical help for all community-dwelling citizens in need of assistance with daily living [24]. Out of 5,4 million citizens living in Norway, 200,000 receive home care services every year [25]. The home care service is organized by municipalities [26], and depending on the size of the municipality, the home care service may be divided into smaller units based on the municipality’s geography. The current study was conducted in Trondheim, Norway, having approximately 200,000 citizens [27]. The temperature in Trondheim typically varies between -20 and + 30 degrees Celsius, and in 2023, 174 days were registered to have rain or snowfall [28].

HCWs working in Trondheim municipality are assistant nurses with high school education, nurses and other college/university health educated personnel (occupational therapists, learning disability nurses, physiotherapists), and assistant healthcare workers without formal health education. Work tasks include direct care tasks (i.e. providing medical or practical assistance to clients) [20, 24], and indirect care tasks (e.g., transportation, documentation) [1, 20]. The allocation of clients to any specific HCW, the work tasks to be performed during each visit, the duration of these tasks, and the transportation mode between client visits, were planned by an operation coordinator in each unit. HCWs were provided this information on a work list during a unit meeting at the beginning of each work shift. The transportation mode determined by the coordinator for a specific HCW depended on the geographical density of the clients assigned to that HCW and the available number of cars in the home care unit.

The present study was conducted in one out of 13 home care units in Trondheim. The geographically restricted district of the home care unit, with height differences up to about 100 m, allowed some use of active transportation (walking, electronic bicycle, or electronic scooter) between clients. Active transportation was mainly used during day shifts, since evening and weekend shifts had fewer direct work tasks and fewer HCWs working, and since every HCW had access to a car during evenings and weekends. If a HCW was assigned an active transportation list, she/he could choose which of the active transportation modes to use.

### Participants

The studied unit was one out of three units in Trondheim municipality where management had agreed to participate with their units as part of a related project, which used a participatory approach to get inputs from home care stakeholders (i.e. HCWs, safety representatives, operation coordinators and managers) on how to redesign home care work for promoting HCWs’ physical health [18]. In that study, one of the suggested redesigns was to instruct the operation coordinator to distribute transportation modes more evenly between HCWs, for the purpose of reducing inter-individual differences between workers in physical activity at work [18]. The specific unit in the present study was chosen among these three since it had a large number of HCWs and did not have an obvious strategy for transportation mode planning. All HCWs in the home care unit who worked at least 50%, had direct contact with clients, and used transportation (i.e. car, walking, electric scooter or electric bicycle) during the workday were invited to participate in a feasibility study (to be reported elsewhere) via email. The first author also held an oral presentation during which the HCWs were invited to participate in data collection. The operation coordinator also informed HCWs of the study during their shift start meetings to ensure that everyone was informed and invited to participate. Participants were excluded from the preceding feasibility study if they had a physical disability hindering normal physical activity, fever and/or sickness on the day of data collection, adhesive tape allergy, and/or were pregnant. All participants in the feasibility study were invited to participate in focus group discussions. The Regional Committee for Medical and Health Research Ethics in Central Norway approved the study (*Project ID: 64,541*), and all participants gave their written informed consent before participation.

### Focus group interviews

Thirty-eight HCWs were invited to participate, where 14 fulfilling the inclusion criteria were available and agreed to take part in focus group discussions. These 14 participants were divided into four focus groups. The groups consisted of three groups with four participants and one group with two participants. The composition and number of participants in each focus group depended on when participants had time available during working hours to participate. The focus group interviews were conducted with the first author (IFL) as the moderator and a research assistant as a co-moderator, following recommendations from Krueger [29]. The interviews were conducted in December 2021 during the HCWs’ working hours at the home care unit facilities. Participants had working time set apart to participate by the operation coordinator. A semi-structured interview guide initially developed to evaluate the feasibility study guided the focus groups (Additional file 1). In short, the focus groups discussed perceived changes during the feasibility study in the planning of transportation modes, any permanent implementation of a more even distribution of different transportation modes between HCWs, and the perceived individual consequences (e.g., for occupational health) of using different transportation modes. The order of questions in the interview guide could be changed depending on how the discussion proceeded. The original guide was pilot tested in a convenience sample of one HCW (master`s student in the project) and one researcher and adjusted when found necessary. On average, the four focus group interviews with the HCWs lasted 51 min, ranging from 43 to 61 min. All interviews were audio recorded.

### Reflexive thematic analysis

The analysis of interviews was inspired by the reflexive thematic analysis described by Braun and Clarke [30, 31] and utilized a manual technique (i.e., printed transcripts and sticky notes). The analysis was guided by the philosophical framework of critical realism and contextualism [30]. Braun and Clark describe the level of semantic or deductive approach in the analysis as placed on a continuum, rather than operating with binary concepts, and that the researcher’s placement on the continuum may change during the analyses [30]. In our analytical process we had a more semantic and inductive approach when writing codes, later shifting to a somewhat more latent and deductive approach to data in close relation to the aims of the research: “*understand HCWs’ perceptions and experiences of using different transportation modes at work, and how they may influence health*”. The latter process was guided by a biopsychosocial understanding of health [32]. This understanding incorporates biological/physical, psychological, and social aspects of health and recognizes that these aspects are interrelated [33]. A biopsychosocial understanding of health is widely accepted and reflected in the definition of health used by the World Health Organization (WHO): “*health is a state of complete physical, mental and social well-being and not merely the absence of disease or infirmity*” [34].

The analysis was led by the first author (IFL) but developed in consultation with the last author (SR) throughout the entire process. The six phases described in 2022 by Braun and Clarke [30] were adopted: First, the interviews were transcribed verbatim and de-identified by IFL, informants being assigned labels in a random order (e.g., I5, I1, I9 instead of I1, I2, I3). The transcribed material comprised 32,164 words in total. Data recordings were listened to several times by authors IFL and SR, and transcriptions were read and re-read while noting initial thoughts that were later discussed. Second, all data were systematically coded by IFL, semantically and inductively condensed to segments of text. Then, codes were refined, collated, and similar codes were compiled. Third, looking across data from the different transcripts, IFL and SR identified shared patterns of meaning that were relevant for the research aims. Relevant codes judged to reflect the same concept were collated and initial themes and subthemes were created. Fourth, themes and subthemes were revised by developing and reviewing the initial themes and subthemes before IFL and SR, in phase five, refined, defined, and finally named themes and subthemes, including reflections by authors MSF and SEM. Finally, in the sixth phase all authors contributed to documenting the results based on a draft written by IFL.

### Reflexivity and trustworthiness

All authors have previous experience with conducting occupational health research in home care. Author IFL is an early career researcher with somewhat limited research experience overall, while authors MSF, SEM and SR are experienced researchers. However, authors MSF and SEM have limited experiences with qualitative research, in particular with regards to the reflexive thematic approach utilized in this study. Braun and Clarke [30] emphasize that reflexive journaling is important when conducting reflexive thematic analysis. In the current study, author IFL performed reflexive journaling throughout the analysis process and tried to reflect upon her previous experiences, assumptions, and beliefs and how this could have influenced the research process and the results. Furthermore, author IFL’s collaboration with author SR was important to ensure that subjectivity was acknowledged, while at the same time keeping a close connection to the data. In writing this paper, we followed the recommendations from the Standards for Reporting Qualitative Research [35], and tried to be as transparent as possible regarding our assumptions and decisions so that readers can, on their own, reflect on the findings’ trustworthiness and transferability [30, 35].

## Results

**Fig. 1:**
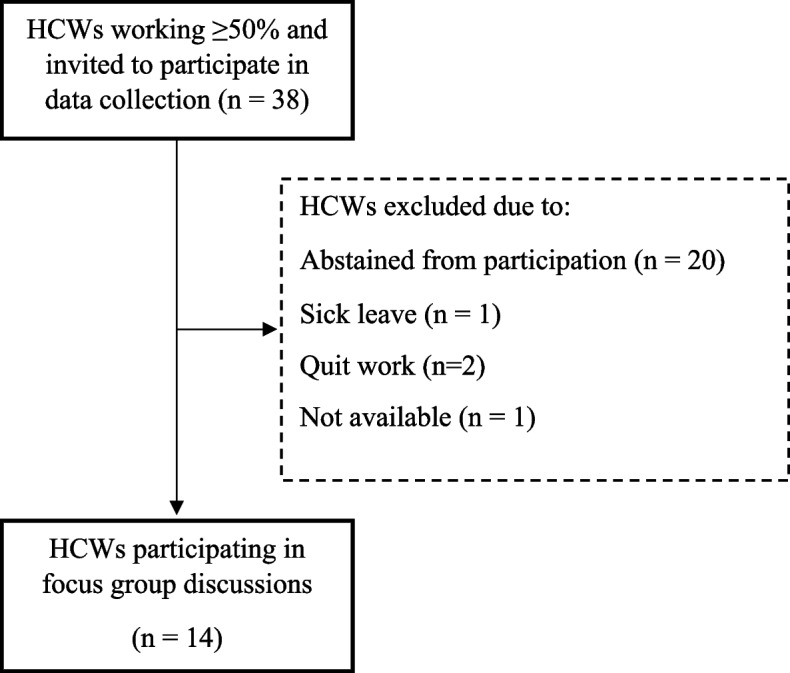
Flow of participants. HCWs: home care workers

Out of 38 invited HCWs, 14 participated in one of the four focus groups. Figure 1 shows a flow chart illustrating the number of HCWs involved in the study and reasons for exclusions.

Characteristics of the 14 participating HCWs are provided in Table 1.

**Table 1:** Participant characteristics (*n* = 14)

Demographic characteristics	N (%)	Mean (SD)
Gender (Female)	11 (79)	
Age (years)		37.4 (13.2)
Work title		
Assistant nurse	5 (36)	
Nurse	5 (36)	
Other^a^	4 (28)	
Seniority in home care (years)		
0–5	8 (57)	
6–10	3 (21)	
18-30^b^	3 (21)	

Values show frequency (percentage), or group mean (standard deviation). ^a^Other health educated personnel with college/university education (e.g., occupational therapist, learning disability nurse). ^b^No participant had seniority in home care between 11 and 17 years

**Fig. 2:**
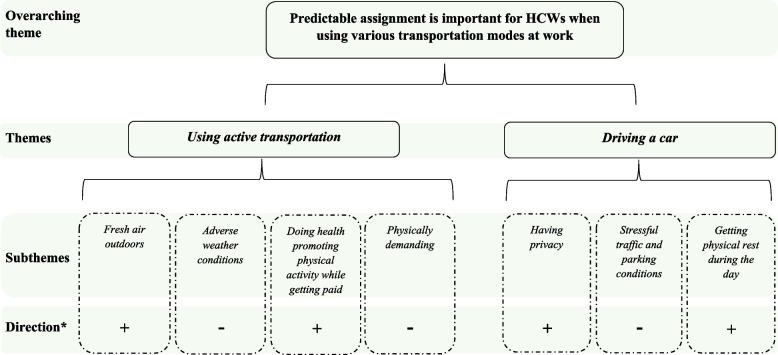
Overview of the hierarchy of themes in the analysis. *Direction on the perceptions and/or experiences related to occupational health. Positive ( +)/ negative (-)

The final analysis arrived at two themes “Using active transportation” and “Driving a car”, and seven subthemes captured within one overarching theme: “Predictable assignment is important for HCWs when using various transportation modes at work”. The structure of the analysis can be found in Fig. 2.

### Overarching theme: Predictable assignment is important for HCWs when using various transportation modes at work

While participants generally perceived their workdays to be unpredictable, they stressed the importance of predictable transportation mode assignments so that unnecessary stress could be avoided, and opportunities to do their jobs could be facilitated. Informant I12 said: “*It's nice to have a bit of variety… as long as there is predictability, it`s fine*”. Informants stressed that the mental loads of the job were more exhausting than the physical and thus that it was important to limit mental stressors so that sick leave and mental burnouts could be avoided. Predictability in transportation mode assignments would make it easier to accept diversity in the assigned transportation mode, and it seemed to be particularly important when transitioning from always driving a car to being assigned an active transportation mode. Informants noted that using active transportation required more planning than driving a car, especially regarding choice of clothes, and indicated the need for information in advance about the transportation mode assignments. For instance, informant I8 said: “*If I'm going to have a day without a car, I'd like to get the info about it so that I can put on some extra clothes*”. While I10 said: “*It’s nice to know if I should wear sneakers or if I should wear boots*”.

Provided that assignments were predictable, many informants believed that using a variety of transportation modes could yield positive health impacts. For instance, one informant accustomed to extensive driving said: *“What can be positive about switching [transportation modes] is that you get a bit more [physical] activity. […] That's nice. And then there's the fact that if you take turns [using active transportation], like us who maybe don't have a single workday all year without having our own car, if we don't have a car that often, then those who don't have a car that often can also get a car… […]”* (I8). Another informant said: “*Variety [from just driving] is nice. You do get a bit fresher from walking outside… a bit more… it can also make you feel more alert*” (I2). Overall, informants generally believed that predictable assignment of walking now and then could have a potential to improve health.

### Theme one: Using active transportation

HCWs had the choice of using electric bicycles, using electric scooters, or to walk for active transportation, but most preferred walking due to concerns about the two electrical transportation modes adding stress to the workday. Stress was due to issues like parking, thefts, and concerns about malfunctions; all representing a mental burden caused by managing these aspects alongside work responsibilities. In this regard informants I2, I5 and I10 discussed: “*It’s more… where to park?*” (I5). “*Yes, that`s a bit of it” (I2).* “*Then there has been theft of bicycles*” (I5). “*There's so much to think about. I have enough to think about without having to focus on that as well*” (I10). Another informants said: “*Sometimes, that [electrical] bike has broken down… So for that reason, I haven`t cycled very much. Because I don't fully trust it*” (I12). Thus, walking emerged as the primarily used mode of active transportation, reflected in their discussions considering only "walking lists" and "driving lists", but not bicycles and scooters. Personal preferences could influence HCWs’ perceptions of walking and some informants thought that this should be considered by the operation coordinator. For instance, one informant said: “*it`s clear that it can be negative [to have to walk] for those who don’t want to walk […]* (I4)”. However, most informants agreed that it was nice to occasionally walk, and talked about several health benefits they believed to acquire from walking, while they, at the same time, reflected upon several negative perceptions and experiences of walking. This is described in further detail in the following subthemes: “*Fresh air outdoors*”, *“Adverse weather conditions*”, “*Doing health promoting physical activity while getting paid*” *and* “*Physically demanding*”.

#### Subtheme: Fresh air outdoors

Walking gave the informants a refreshing break and a connection to the outdoors, serving as a contrast to the intense situations they faced inside clients’ homes. For instance, informants I12 and I6, who were in the same focus group, discussed the satisfaction of walking after client shower assignments: “*It`s good to walk. […]*” (I12). “*For me, the same… We have shower duties, so if I have one and two showers a day, it`s fresh air after the showers*” (I6). “*Yes*” (I12). “*When I drive, there is heating… but if I go out to walk: aah, fresh air […]. So I think it`s very lovely*” (I6). Similarly, I9 said: “*To actually unwind a little bit after visiting someone… because with some you… It's pretty intense. Where you stand for maybe an hour, in a shower. And then to just come out afterwards*”. Walking and experiencing fresh air during the workday appeared to alleviate mental exhaustion better than driving. Informant I3 said: *When I have walking lists, I feel less tired […]. Yes, you get some fresh air during the day then. I think it's nice at least*”. Moderator: “*Are you less tired physically or mentally, or both?*”. I3: “*Mentally, actually*”.

#### Subtheme: Adverse weather conditions

While informants appreciated the positive mental effects of fresh air and visits to the outdoors, they also emphasized that this was, to some extent, dependent on the weather and daylight conditions. Bad weather was a big topic of conversation for the informants when talking about walking, with frequently occurring statements like: “*I like to have walking lists in the summer, but I don’t like it in the winter* […]” (I2), “*Obviously, no one wants to walk when it’s minus 25[degrees celsius]*” (I14) and “*It’s the weather then. We're outside in all kinds of weather, and it pours rain and suddenly it snows. And then it's slippery and then…*” (I7). “*… the northwest wind blows.* (I10)”. Informant I10 talked about a bad experience with walking in rainy weather and said: “*[…] then my socks were soaking wet from 11 to three. So, I could have gotten sick and not come to work the next day, so to speak”.* The absence of a shelter for writing and reading reports between visits reenforced the discomfort experienced during bad weather, and informants talked about having to stand outside or sit in cold staircases in apartment buildings. Walking at night, in the dark, was also emphasized as being associated with feeling unsafe and anxious. Thus, having to walk between clients’ homes led to increased risks of freezing and maybe getting sick, and/or being scared. It also increased the risk for injuries due to slips and falls, especially during winter. Walking between clients’ homes also meant additional time spent on dressing and undressing, leading to time pressure and stress, which was avoided when using a car: “*You spend so much time on dressing and undressing [yourself]… But you just have to* [*…*]” (I10). Overall, HCWs perceived that bad weather conditions posed challenges and increased health risks during walks between visits.

#### Subtheme: Doing health promoting physical activity while getting paid

Overall, the informants saw transportation time as an opportunity for themselves to engage in healthy physical activity. They believed that using active transportation, especially walking, was a good way of getting regular physical activity, which helped them to keep in shape and was health-promoting. Statements on this were common, like “*[walking means that one] gets in shape”* (I5), “*[when you have a walking list] you do achieve 10,000 steps, so to speak. […] That's a good thing*” (I2) and “*[walking] has an effect on our health. […] I would think that one stays a bit healthier and quicker when one walks*” (I12). Some informants even joked about being so fortunate to have the opportunity to exercise while getting paid. Getting a walking list now and then was emphasized as being particularly important for the less physically active HCWs as they would get the largest health benefits from walking. Many informants described a need for movement and engaging in various forms of physical activity after assisting clients in activities of daily living, such as dressing or showering. For instance, informants I4 and I6 who were in the same focus group said: “*Just being able to walk and use your body*” (I4). “*Not least!!*” (I6). *“…after you might have been in a care where you have been a bit… in uncomfortable positions, then you get out and move a bit*” (I4). “*Yes!*” (I13). “*…in a different way*” (I4). In these cases, informants had experienced that walking was better for them than sitting down in a car.

However, a few informants disagreed that having walking lists at work would influence their health. For instance, one informant said: “*[…] You do things in your spare time [that is health promoting]. And to walk a day at work, I don’t think… I don’t think it would make much of a difference to me [and my health] at least*” (I11).

#### Subtheme: Physically demanding

While informants recognized the positive health impact of walking during work, they also emphasized that only having walking lists could lead to physical exhaustion, because the total physical load became too high over time, and opportunities for physical rest were limited. Informant I13 said: “*But what can be a bit negative [with walking lists] is that there can be a lot of walking. So, you can get quite tired. […] on a normal weekday I can walk 7–8 km. And then I feel it at the end of the day […] Then I start to get tired”*. Informants reported walking between 10,000 and 13,000 steps per shift when they had walking lists, which they found physically demanding in the long run. Even those who appreciated walking lists acknowledged the value of sometimes having a car for work, which offered more rest. Informant I2 elaborated: “*There's a lot of standing during the day if we don't have the opportunity to sit down, like you do in a car. So, I notice that I'm more exhausted after a day with a walking list than I am with a car list*” (I2). Some HCWs, particularly older workers with physical health issues, could also find it physically demanding to walk between clients' homes. For these HCWs, informants believed that getting physical rest between assignments could be vital for their ability to continue working as HCWs.

### Theme two: Driving a car

Overall, informants talked in more positive terms about driving than about walking, highlighting it as a chance to get both psychosocial and physical rest on the journey between clients’ homes. They considered the car to be a private space allowing them to process and recuperate from the mental and physical demands encountered during their workday. However, the quality of rest and recovery in the car was affected by traffic and parking conditions. This is expanded upon in the upcoming subthemes: “*Having privacy*”, “*Stressful traffic and parking conditions*” and “*Getting physical rest during the day*”.

#### Subtheme: Having privacy

Having a car was described as providing a private space for the HCWs during their workday. This was a space both for processing mental impressions accumulating during the workday, as well as for getting a break, for which opportunities were otherwise few. The car was the only space where the informants could be alone during the day, and take some time, even for just a minute, to react and process difficult experiences before rushing to the next client. This appeared when informants said things like those discussed by I7, I2 and I10 who were in the same focus group: “*You often hear that someone has sat and cried a bit in the car and such, between the clients*” (I7). […]. “*Yes, it can be quite a lot. Many clients to deal with. Many clients struggle mentally, you know. And then you are alone in it, and then there`s not much room to…[process it]”* (I2). “*No, and then we don't have time to feel it either, in between…”* (I7). “*No*” (I2). “*You kind of don't have time to sit down for a bit by yourself and maybe sort of*…” (I7). “*Process it a bit*” (I10). Informants noted the lack of private spaces while walking, feeling more exposed and visible then.

Also, the car offered a mental time-out by, for instance, listening to the radio. In this regard, informant I12 said: “*Radio is nice. […] So it’s quite nice to listen to news or the radio when driving. That short distance you have*”.

#### Subtheme: Stressful traffic and parking conditions

Stressful traffic conditions could diminish the restorative effects of driving a car. Challenges like traffic congestion and parking difficulties increased stress and time pressure for the informants. For instance, informants said: “*Parking. […] Finding a legal parking spot, at least nearby [can be difficult]*” (I9). “*Then you have to watch the parking meter and look after this and that, and it takes some time to arrange in the app […]”* (I5). “*And then on hectic days not finding parking, it`s very stressful*” (I6). Difficulties finding and paying for suitable parking sometimes led to illegal parking, which could result in a parking ticket, and subsequent personal expenses for the HCWs, as mentioned by informant I4: “*If we get a fine, we have to pay it ourselves*”.

#### Subtheme: Getting physical rest during the day

Irrespective of the transportation mode, informants perceived their job as physically demanding, involving considerable walking and standing. Consequently, many stressed the importance of sitting down whenever possible, and considered access to a car crucial in allowing moments of rest. Even informants who enjoyed physical activity acknowledged the benefit of cars. For instance, informant I2 said: “*I must honestly admit […]… even though I`m very conscious that one should be active and so on… I'm really fond of that car and the opportunity to sit down […]*”. I10 replied: “*I think it's about the total burden again*”.

## Discussion

The aim of this study was to understand HCWs’ perceptions and experiences of using different transportation modes at work, and how they may influence health. To our knowledge, this is the first study to investigate this topic. The main results of the analyses can be condensed into “*Predictable assignment is important for HCWs when using various transportation modes at work*”. Most HCWs believed that walking now and then could lead to positive health impacts compared to only driving a car. However, it appeared important to assign transportation modes in advance, so that they were predictable for the HCWs. This was reported by the HCWs to decrease unnecessary stress. Some informants questioned whether employing varied transportation modes would be health-promoting for all HCWs, emphasizing that some individual considerations should be made regarding high age, physical health challenges or strong preferences in what transportation mode to use.

Informants in the current study stressed that the mental work-related strain was more exhausting than the physical load, and that reducing mental stressors was important to avoid sick leave and mental burnouts. Thus, the current study indicates that biopsychosocial aspects of health should be considered when assigning HCWs to a variety of transportation modes. At the same time, this study illustrates that different aspects of health may be in conflict [33]. Several aspects, both beneficial and adverse, were highlighted for both walking and driving. While walking now and then was perceived to be health-promoting, bad weather conditions could increase the risk of getting sick and encounter injuries due to slips and falls. This has been mentioned as a potentially negative health factor for HCWs in previous literature as well [7, 36]. Weather conditions also affect mobile healthcare workers (e.g. social workers, physicians and HCWs) while driving a car, including feeling unsafe when driving in snow and unpredictable weather [17]. This may lead to increased feelings of stress [17]. In the current study, walking too frequently was deemed “*physically demanding*” and did not offer sufficient opportunities for rest, which can be beneficial for workers in physically demanding occupations [37, 38]. Driving, on the other hand, was generally perceived as the more attractive mode of transportation, despite several informants mentioning that congested traffic and problems in finding parking spaces could lead to stress. Having to drive long distances has also been emphasized as tiring by mobile healthcare workers, while at the same time giving the worker a mental break [17]. This was supported by the findings in the current study, where HCWs perceive driving a car as an opportunity to have privacy during the workday.

HCWs have previously been described to work in unpredictable work environments [7]. A key finding in this study is that introducing a planning of transportation mode in advance so that it is predictable to the HCWs may alleviate stress and facilitate their opportunities to do a good job. Predictability seemed to be particularly important when transitioning from always driving a car to being assigned active transportation modes, since this required more planning on the part of the HCWs, especially regarding what clothes to wear.

Informants in the present study stated that some HCWs, like those being older, may require more rest than others, and that they should be assigned less active transportation. This is in line with a study by Pohjonen [39], arguing that physical work demands among HCWs should be reduced for aging workers, since work ability decreases with age. However, the health benefits of physical activity, such as walking and cycling for transportation, have been well documented [40], and having HCWs of higher age engaged in more health-promoting physical activity might enhance their capacity and help them to stay longer at work with a good health.

Some informants emphasized that strong individual preferences regarding transportation modes should be acknowledged when assigning transportation. Possibilities to control one's own work is a predictor of perceived work ability among HCWs [39], and the level of autonomy may influence work engagement, intentions to stay in health care and the likelihood of burnouts [41, 42]. Thus, the transportation mode assignment may represent a trade-off between promoting the psychosocial work environment if using a car, and potential long-term enhancement of capacity and physical health if using active transportation. The idea of balancing elements of work to offer a healthy “just right” trade-off between load and recovery is expressed in a newly developed approach to occupational health: Goldilocks Work [43, 44], as well as in more established models such as the Job Demand – Resources model of Burnout [45].

### Implications for practice and further research

Overall, the findings in the present study indicate that HCWs perceive walking lists as a health-promoting measure, if they are used now and then. However, home care managers and researchers that intend to implement transport initiatives similar to those described here should secure that transportation modes are predictable at a daily basis for the HCWs. Management should also consider the age of HCWs when assigning transport modes, and possibly introduce preference-based planning to some extent. Researchers should investigate short- and long-term effects in both physical and psychosocial health in different age groups of using a variety of transportation modes in home care units, compared to not varying the modes. Further research is even needed in other occupations involving extensive outdoor transport, such as mail services, in order to understand the extent to which the present findings are specific to HCWs.

### Study limitations

The current study has some limitations. First, the focus groups were small, with one group comprising only two participants. An ideal size in most focus group settings would be four to eight participants per group [29], but this was not feasible in all the present groups since participants had to perform care tasks at different times. We chose to include the focus group with only two participants, as we considered their perceptions and experiences to be just as important as those of the other participants. Second, the study was carried out in one home care unit in one urban Norwegian municipality, likely limiting transferability of the results, especially regarding the effects of contextual factors such as traffic and weather conditions. The region's harsh, lengthy winters specific to the studied region were a frequent discussion point in the focus groups. Readers should therefore judge transferability of the results to other contexts based on the contextual information provided [29, 30]. Third, although no comparison between different job titles were performed in the current study, some findings indicated a possible difference between nurses and other HCWs. Apprehending these differences further, for instance in terms of living conditions during leisure, could have provided interesting information in the study. For instance, a previous study by Jackson et al. [17] on mobile healthcare workers showed that education and job title influenced experiences of mobile healthcare work.

## Conclusions

Walking now and then between client visits was generally believed by the HCWs to lead to positive health impacts compared to only driving a car. Introducing planning of various transportation modes in advance so that they are predictable to the HCWs seems to be an important measure to reduce stress among HCWs. In addition, some individual factors (age, physical health challenges or strong preferences) should be considered in the planning, and it should be realized that the planning likely represents a trade-off between promoting the psychosocial work environment when driving a car and potentially enhancing long-term physical health when using active transportation. Thus, biopsychosocial aspects of health should be considered when planning the mode of transport between client visits for HCWs.

## Supplementary Information

Additional file 1. Guiding Questions for the HCW Focus Groups: Themes (capital letters), opening questions (regular), key questions (bold) and sub questions (italic).

## Data Availability

The dataset used during the current study can be available from the corresponding author on reasonable request.

## Abbreviation

HCW: Home care worker
