# Supplementary material for: On the move: understanding home care workers’ experiences of using various modes of transportation at work in an occupational health perspective
Source: BMC Health Serv Res. 2024 Dec 18;24:1565. doi: 10.1186/s12913-024-12071-z (PMC11653748; doi:10.1186/s12913-024-12071-z)
Supplement: Supplementary file 1 — Additional file 1. Guiding Questions for the HCW Focus Groups: Themes (capital letters), opening questions (regular), key questions (bold) and sub questions (italic). [file 12913_2024_12071_MOESM1_ESM.docx]

**Additional file 1**

Guiding Questions for the HCW Focus Groups: Themes (capital letters), opening questions (regular), key questions (bold) and sub questions (italic)

| Theme | Opening question |
| --- | --- |
|  | What is your first name, work title and duration of employment in Home Care? |
| PERCIVED CHANGES IN TRANSPORTATION MODE PLANNING | **Can you describe how you perceived the distribution of planned transportation use at work the four weeks of intervention?** |
|  | *What has been different?* |
|  | *Did anything function better during this period? What?* |
|  | *Did anything deteriorate during this period? What?* |
|  | *What are the advantages of using car/walking/electric bicycles/electric scooter at work?* |
|  | *What are the disadvantages of using car/walking/electric bicycles/electric scooter at work?* |
|  | *Did anyone of you used other means of transport than what you are used to? How was that?*  *When you were planned to use active transport, did you use active transport? Why/why not?*  *Have you experienced anything in the last four weeks that has changed your opinion about using different transportation modes? What? Why?*  *Do you have any thoughts about whether your work is/was affected by type of transport used?*  *Is there anything we could have done to achieve a better distribution of walking/driving lists?* |
| PERMANENT IMPLEMENTATION | **Let’s say that this measure (applying a variation in transportation mode use for all employees) would be induced on a permanent basis. What do you think about that?** |
|  | *Let’s say that one included use of active transport also on evening and weekend shifts. What do you think about that?* |
| PERCIVED INDIVIDUAL CONSEQUENCE | **Which consequences do you think that using different transportation modes during work can have for you and your colleagues?** |
|  | *How and why?* |
|  | *Regarding individual health?* |
| ENDING THE INTERVIW | *Regarding productivity at work?*  *Do you have anything else you would like to address or does anyone feel that there is something they have not been able to say?*  *Thank you for participating* |
